# The SAR11 Group of Alpha-Proteobacteria Is Not Related to the Origin of Mitochondria

**DOI:** 10.1371/journal.pone.0030520

**Published:** 2012-01-23

**Authors:** Naiara Rodríguez-Ezpeleta, T. Martin Embley

**Affiliations:** Institute for Cell and Molecular Biosciences, Newcastle University, Newcastle upon Tyne, United Kingdom; Université Paris Sud, France

## Abstract

Although free living, members of the successful SAR11 group of marine alpha-proteobacteria contain a very small and A+T rich genome, two features that are typical of mitochondria and related obligate intracellular parasites such as the Rickettsiales. Previous phylogenetic analyses have suggested that *Candidatus* Pelagibacter ubique, the first cultured member of this group, is related to the Rickettsiales+mitochondria clade whereas others disagree with this conclusion. In order to determine the evolutionary position of the SAR11 group and its relationship to the origin of mitochondria, we have performed phylogenetic analyses on the concatenation of 24 proteins from 5 mitochondria and 71 proteobacteria. Our results support that SAR11 group is not the sistergroup of the Rickettsiales+mitochondria clade and confirm that the position of this group in the alpha-proteobacterial tree is strongly affected by tree reconstruction artefacts due to compositional bias. As a consequence, genome reduction and bias toward a high A+T content may have evolved independently in the SAR11 species, which points to a different direction in the quest for the closest relatives to mitochondria and Rickettsiales. In addition, our analyses raise doubts about the monophyly of the newly proposed *Pelagibacteraceae* family.

## Introduction

According to the endosymbiotic theory, mitochondria and related organelles such as hydrogenosomes and mitosomes arose from an endosymbiotic bacterium [1], [2], [3]. Phylogenetic analyses based on mitochondrial-encoded proteins have well established that this bacterium was a member of the alpha-proteobacterial division [4], [5], and although the exact position of the mitochondrion within the alpha-proteobacteria is still debated [6], most of the trees published place the mitochondrial ancestor within or as sister group to the intracellular obligate parasitic Rickettsiales [7], [8], [9], [10].

Within the alpha-proteobacteria, the SAR11 lineage of free-living marine organisms has received great attention since its discovery in the early 1990s for being among the most successful organisms on the planet [11], [12]. Phylogenetic trees based on four bacterial encoded proteins place *Candidatus* Pelagibacter ubique, the first cultured organism of this group, within a cluster of alpha-proteobacteria that excludes Rhodospirillales and Rickettsiales [12], whereas another study, based on 16 bacterial/mitochondrial proteins places this species as sister group of the mitochondria/Rickettsiales clade [9]. Recently, the controversy has been revigorated up by four other studies, published almost simultaneously, that support alternative placements for the SAR11 group: Thrash *et al*. [13] and Georgiades *et al*. [14], who conclude that the SAR11 group belongs to the mitochondria/Rickettsiales clade, and Brindefalk *et al*. [15] and Viklund *et al.* [16], who argue that these two groups are not related.

**Figure 1:**
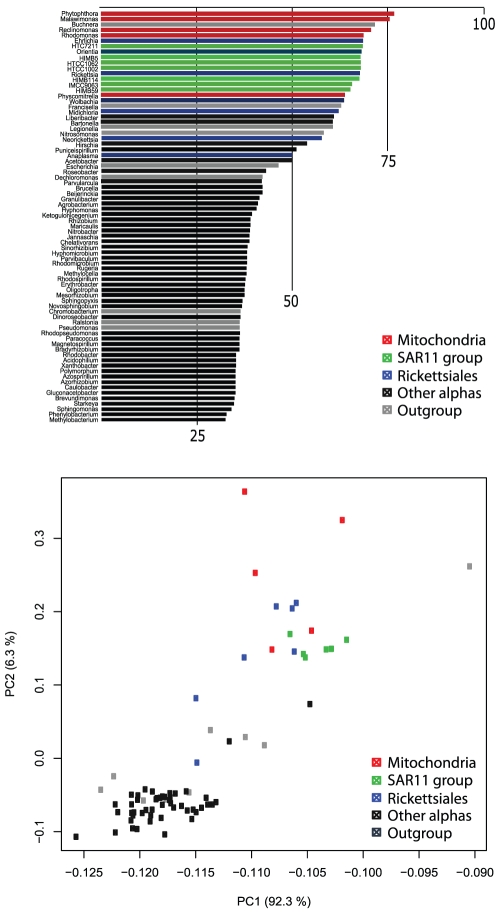
Nucleotide and amino acid composition of the dataset used in this study. A) Bar graph displaying the percentage of A+T in the concatenation of the nucleotide sequences. B) Reduced dimensionality plot showing the main principal components of the global amino acid compositions. Dots are coloured as in Figure 1A. The group of overlapping dots at the bottom right corner contains the species with the lowest A+T %. The variances that explain the two first axes are respectively 92.3% and 6.3%.

From the four studies, only Thrash *et al* [13] include more representatives of the SAR11 group other than *Ca.* Pelagibacter ubique in analyses based on several genes, the others including only this species to represent the whole group. Moreover, Brindefalk *et al* [15] and Georgiades *et al* [14] use a single species (*Reclinomonas americana*) to represent mitochondria in their analyses based on several genes, and Viklund *et al* [16] does not include this organelle in their inferences. On the other hand, Thrash *et al* [13] and Georgiades *et al* [14] did not use sophisticated models of sequence evolution that take compositional bias into account, as did Viklund *et al* [16], and Brindefalk *et al* [15]. Finally, the conclusions drawn from some of these studies are derived from combining modestly supported results [13] or from deciding on one of the contradictory outcomes obtained (see Figures 1 and S2A of Viklund *et al.* 2011).

Therefore, in order to settle the controversy on the position of the SAR11 group within the alpha-proteobacteria and its relation to the origin of mitochondria, phylogenetic analyses based on a broad taxon sampling, including more available members of the SAR11 group, and using sophisticated models of sequence evolution are necessary. Here, we have assembled a dataset of 24 evolutionarily conserved orthologous proteins from 5 mitochondria, 62 diverse alpha-proteobacteria, including 6 members of the SAR11 group, and 9 other bacteria as outgroup. Our results confirm that the SAR11 group does not belong to, nor is sister group of, the mitochondria/Rickettsiales clade.

## Results and Discussion

### Phylogenies based upon nucleotide data

Shared high percentage of A+T in rickettsial and mitochondrial genomes has been blamed for the difficulties in confidently identifying the closest relatives to michochondria [8], [10]. This shared nucleotide composition is illustrated in Figure 1A, where the high proportions of A+T of mitochondrial and rickettsial genomes are noticeable; the same pattern is also observed in members of the SAR11 group. Remarkably, in a phylogenetic analysis based on nucleotide data, the effect of the compositional bias is so great that the assemblages obtained seem to reflect nucleotide composition more than evolutionary relationships; for example, the monophyly of the outgroup not recovered (Supporting Information S1 and S2). Nucleotide compositional bias is a well documented source of phylogenetic inference artefacts that, if not taken into account in the evolutionary model used, may negatively influence tree reconstruction [17]. The RY coding [18], which consists on grouping purines (A&G) and pyrimidines (C&T) in two character states (R and Y) prior to phylogenetic inference, can sometimes reduce the effect of compositional bias [19], [20]. In our case, this approach has a positive effect on the obtained tree (Supporting Information S3), which looks more consistent with current ideas for alpha-proteobacterial phylogeny [21] than the tree built using the four character states; additionally, it strongly supports the grouping of mitochondria, rickettsiales and the SAR11 group. This result is in accord with the conclusions of Williams, Sobral, and Dickerman [9], Thrash *et al*. [13] and Geogiades *et al*. [14].

### Phylogenies based upon amino acid data

**Figure 2:**
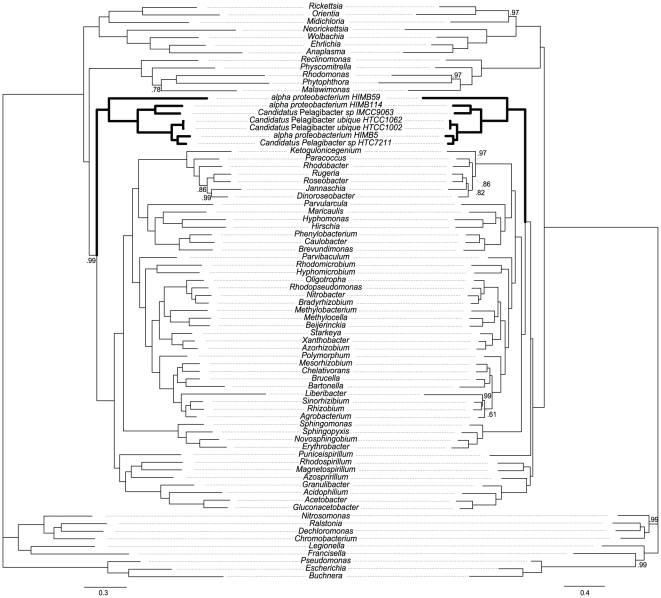
Phylogeny based on 24 mitochondrial/bacterial proteins (6,542 amino acid positions) inferred by Bayesian Inference with the WAG + F (left) or the CAT mixture (right) model. Numbers indicate posterior probability values. Branches without values are supported by posterior probabilities of 1.0. The scale bar denotes the estimated number of amino acid substitution per site. See supplementary material for complete species names and proteins used.

Biases in nucleotide proportions affect amino acid composition [22], an effect that is also observed in our dataset (Figure 1B). Yet the phylogeny obtained with amino acid data is not as biased as the one obtained with nucleotide data and recovers all major alpha-proteobacterial groups and the relationships within them using both Bayesian (Figure 2) and ML inferences (Supporting Information S4). This is expected given that amino acids generally have more character states and are therefore less prone to homoplasy than nucleotides. In the Bayesian tree constructed based on the WAG matrix for amino acid substitution, mitochondria and Rickettsiales are not monophyletic nor are any of them are related to the SAR11 group. Interestingly, applied to our dataset, the site heterogeneous mixture model CAT [23] drastically changes the position of the SAR11 group, which is now placed within a cluster of alpha-proteobacteria that excludes Rickettsiales and Rhodospirillales. Additionally, the sister-group relationship of mitochondria and Rickettsiales is strongly supported in this case (Figure 2).

**Figure 3:**
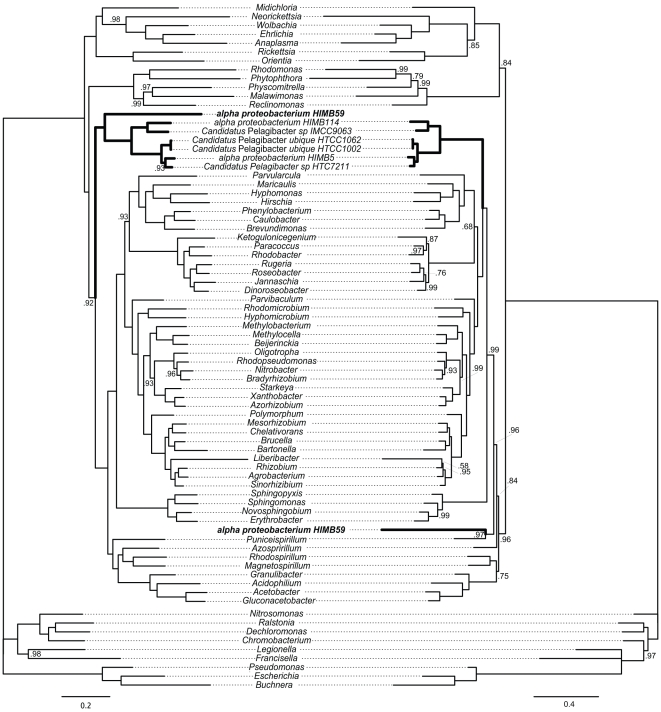
Phylogeny based on 24 mitochondrial/bacterial proteins (6,542 amino acid positions) inferred by Bayesian Inference with the GTR + F (left) or the CAT mixture (right) model on the Dayhoff recoded dataset. (see Methods for details). Branches without values are supported by posterior probabilities of 1.0. The scale bar denotes the estimated number of amino acid substitution per site. See supplementary material for complete species names and proteins used.

Analogous to the RY coding for nucleotides, the Dayhoff coding in functional categories has been proposed to reduce amino acid compositional biases [24]. Applied to our dataset, this method does not have a remarkable effect in the tree based on the WAG matrix (Figure 3 and Supporting Information S5). However, when the CAT model is employed on the Dayhoff recoded dataset, an even more drastic change in the position of the SAR11 group is observed: all members of the group but one are placed as a monophyletic assembly in a cluster that excludes Rickettsiales, Rhodospirillales and Sphingomonadales (Figure 3). Surprisingly, alpha proteobacterium HIMB59 is not placed within the SAR11 in this case and appears related to the SAR116 group member *Candidatus* Puniceispirillum IMCC1322.

### Alpha proteobacterium HIMB59 may not be a member of the SAR11 group

**Figure 4:**
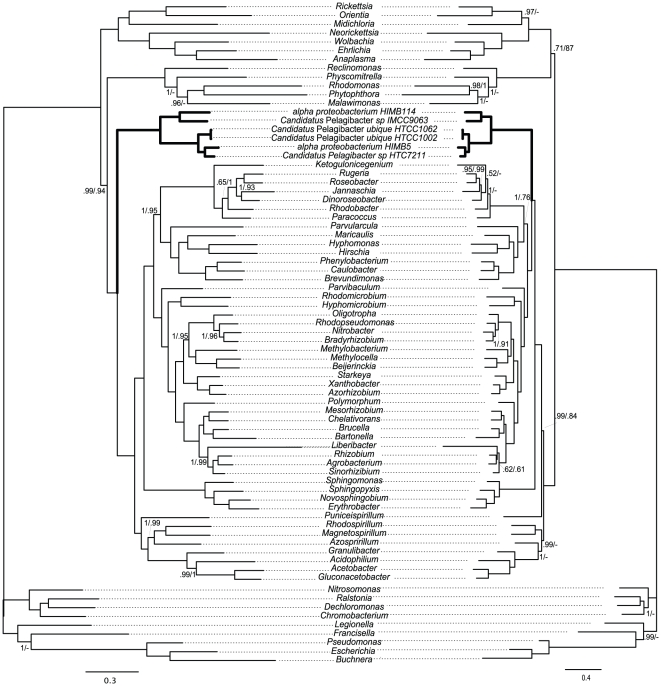
Phylogeny based on 24 mitochondrial/bacterial proteins (6,542 amino acid positions) inferred by Bayesian Inference with the WAG/GTR + F (left) or the CAT mixture (right) model on the non recoded and Dayhoff recoded dataset. (see Methods for details). Branches without values are supported by posterior probabilities of 1.0. When at least one dataset gives posterior probability <1, both values are shown, standard coding on the left and Dayhoff coding on the right. The scale bar denotes the estimated number of amino acid substitution per site. See supplementary material for complete species names and proteins used.

In order to further understand the evolutionary position of alpha proteobacterim HIMB59 and the effect of the inclusion of this species on the position of the SAR11 group, we performed phylogenetic analyses excluding this taxon or having it as the sole representative of the SAR11 group. As shown in Figure 4, excluding HIMB59 makes the result from the non recoded and recoded datasets more similar to each other, specially in the case of the CAT mixture model, where both datasets place the SAR11 in a cluster that excludes Rickettsiales, Rhodospirillales and Sphingomonadales. In turn, when only HIMB59 is included to represent the SAR11 group, this species maintains the evolutionary position observed in Figures 2 and 3 under both models and data recodings used (Supporting Information S6, S7, S8 and S9). This results suggests that HIMB59 may not be a member of the SAR11 group and that the definition of the family *Pelagibacteraceae*, fam. nov. recently proposed by Thrash *et al*. [13], needs to be revisited. Indeed, in their analyses, Thrash *et al*. [13] also observed some instability of HIMB59, which in some cases branched with mitochondria.

### Taxon sampling and compositional bias affect the positioning of the SAR11 group and of HIMB59 in phylogenetic trees

Among the competing alternative positions for the SAR11 group observed in our analyses based on amino acid data, none supports the conclusions of Williams, Sobral, and Dickerman [9], Thrash *et al*. [13] and Geogiades *et al*. [14], who suggested a common ancestor of mitochondria and the SAR11 group. This is also true when using %AT rich or poor outgroups (Supporting Information S10 and S11). Only the tree based on nucleotide data points to this direction, implying that the sister group of mitochondria/Rickettsiales with the SAR11 group may be the result of a tree reconstruction artefact caused by compositional bias. Indeed, as we apply methods to correct for compositional bias such as Dayhoff recoding or use sophisticated models of evolution that take site-specific compositional heterogeneity into account such as the CAT model, the SAR11 group gets further and further away from mitochondria and Rickettsiales and branch deeper in the alpha-mitochondrial tree. This may indicate that the true evolutionary position of the SAR11 group is within a group of alpha-protebacteria that excludes Rickettsiales, Rhodospirillales and Sphingomonadales, but that the shared compositional bias of mitochondrial, Rickettsiales and the SAR11 sequences causes the SAR11 group be attracted to the other two groups. This idea is supported by the analyses of Viklund *et al.* [16], who pointed to a compositional bias as responsible for the traditional relationship of *Pelagibacter* with Rickettsiales. Similarly, the positioning of HIMB59 within the SAR11 group may also be caused by an artefact due to shared amino acid composition between this taxa and the SAR11 group (see Figure 1B).

### Outlook

The genome of *Candidatus* Pelagibacter ubique is among the smallest to replicate independently and has an extremely high percentage of A+T content [12], two features that are often observed in obligate intracellular parasites such as Rickettsiales [25], but which are more surprising in a free-living marine organism [26]. These two features together with the phylogenetic analyses of Williams, Sobral, and Dickerman [9] have lead some authors to label *Pelagibacter* as the closest free-living outgroup to Rickettsiales [13], [27], [28], implying that mitochondria diverged from the alpha-proteobacteria at some time between the divergence of a marine clade and a strictly intracellular lineage adapted to various eukaryotes.

The analyses presented here suggest however that the SAR11 group is not specifically related to the Rickettsiales, and that genome reduction and bias toward a high A+T content have evolved independently in both lineages. Indeed, it has already been suggested that a reduction in genome size, which is often accompanied by an increase in A+T content, may be a selective advantage in the open ocean where nutrients are scarce [26]. This may explain the presence of these exceptional parasite-type features in *Pelagibacter* and why this species belongs to one of the most successful groups of organisms in the planet. Additionally, some features common to all Rickettsiales that are not present in *Pelagibacter* and vice versa support the view that those two lineages are not related. For example, Rickettsiales contain an unusual histidyl t-RNA synthetase that is not present in *Pelagibacter* [29], and *Pelagibacter* contains a particular type IV secretion system [30], a 2/2 Hb1 globin [31], a unique glycine activated and a SAM-V riboswitch [32], [33], and a signal recognition particle protein [27] that are not present in Rickettsiales.

In conclusion, our analyses based on a broad taxon sampling including several members of the SAR11 group are consistent with the current view that Rickettsiales are the closest relatives to mitochondria, but they do not support a close relationship of *Pelagibacter* and the SAR11 group to the origin of this clade. Therefore, if the potential relationship of SAR11 to mitochondria has been used as an argument to support the search for mitochondrial related organisms in marine environments [9], [13], the alternative placement of SAR11shown here should also encourage research focused on how genome reduction evolves in free-living organisms.

## Methods

### Dataset construction

Starting from the 67 protein coding genes of the *Reclinomonas americana* mitochondrial genome, BLASTP searches were performed on the 539 complete alpha-, beta- and gamma-proteobacterial genomes available as of September 2011 in GenBank, on 5 slowly evolving mitochondrial genomes and on five additionaly SAR11 group genomes retrieved form GenBank (Candidatus *Pelagibacter ubique* HTCC 1002, Candidatus *Pelagibacter* sp HTCC7211 and alpha proteobacterium HIMB114) and from the JCVI (HIMB5 and HIMB59). All protein sequences with a Blast e-value lower than 10^−4^ were retrieved. Each set of sequences was aligned at the amino acid level with Muscle [34], manually refined with ED [35], and trimmed of unambiguous aligned blocks of positions with Gblocks [36] with the following parameters: a minimum of 50% and 75% of sequences identical for a conserved or flanking position respectively and a maximum of 5 contiguous non conserved positions and a minimum of 5 positions for a block. Selected blocks were manually verified and introduced in the dataset if missing data was responsible for the automatic removal. Nine beta- and gamma- proteobacteria representing a broad range of %GC content were selected as outgroup, and 62 proteobacteria were selected by selecting one species per genera and by removing the ones that had more than 50% of missing data (Supporting Information S12). The 24 genes that contained at least three eukaryotes and that lacked at most 14 of the 76 selected species were retained for further analyses (Supporting Information S13). Once orthologous sequences selected with SCaFoS [37], new alignments were performed with Muscle and trimmed with Gblocks (same parameters as above) at the protein level. The corresponding nucleotide sequences were extracted using in house software, and alignments, including trimmed sites, were matched to the protein datasets with Revtrans [38] and in house software. The concatenation of the 24 protein coding genes comprises a total of 6,542 amino acids and 19,626 nucleotide positions. 7% of the data is missing (see Supporting Information S12 and S13). The amino acid composition bias of the taxa in the dataset was visualized by assembling a 76×20 matrix containing the percentage of each amino acid per species using the NET program [35]. This matrix is displayed as a two dimensional plot in a Principal Component Analysis (PCA).

### Phylogenetic analyses

The concatenated protein dataset was analyzed by Maximum Likelihood, with RaxML [39], and Bayesian Inference, with Phylobayes [40]. The dataset was analyzed at the nucleotide and amino acid level in both, standard coding (4 or 20 character states respectively) or as purine/pryrimidine (RY) coding or Dayhoff coding of amino acid in six functional categories [24]. To allow for a general-time-reversible (GTR) matrix implemented in RaxML, the dataset was recoded to four categories instead of six, by combining aromatic (FYW) and hydrophobic (MVIL) amino acids and coding the rare cysteine as missing data [41]. For the Phylobayes analyses, the 6 Dayhoff categories were considered. RaxML and Phylobayes analyses were performed with the WAG (for standard amino acid coding) and GTR (for the nucleotides and Dayhoff4 or Dayhoff6 coding) matrices, gamma distributed rates across sites (4 discrete categories) and stationary nucleotide or amino acid frequencies estimated from the dataset (WAG/GTR+F+**Γ4** model). Phylobayes analyses were also performed with the CAT mixture model that accounts for site-specific compositional heterogeneity [23]. See Supporting Information S14 for details on the Phylobayes analyses (WAG, GTR-Dayhoff6, CAT, CAT-Dayhoff6). Cross validation analyses as implemented in Phylobayes 3.3 were performed in order to select the best fitting model (Supporting Information S15), which was the CAT +**Γ4** for both, non coded and recoded amino acid data. Posterior predictive tests for saturation and compositional homogeneity were also performed and showed that, as expected [42], the CAT model better estimates saturation (Supporting Information S16) and the Dayhoff recoded dataset's heterogeneity is better accounted for than that of the non recoded dataset for the GTR matrix but not for the CAT model (Supporting Information S17).

## Supporting Information

Supporting Information S1 **Phylogeny based on 24 mitochondrial/bacterial protein coding genes inferred by Maximum Likelihood using GTR+F+Γ4 model.** Values above branches indicate bootstrap values. The scale bar denotes the estimated number of nucleotide substitution per site. Bars on the right represent the A+T percentage and are proportional to those in Figure 1.
(TIF)

Supporting Information S2 **Phylogeny based on 24 mitochondrial/bacterial protein coding genes excluding the third codon position inferred by Maximum Likelihood using GTR+F+Γ4 model.** Values above branches indicate bootstrap values. The scale bar denotes the estimated number of nucleotide substitution per site. Bars on the right represent the A+T percentage and are proportional to those in Figure 1.
(TIF)

Supporting Information S3 **Phylogeny based on 24 mitochondrial/bacterial protein coding genes inferred by Maximum Likelihood using the RY coding and the GTR+F+Γ4 model.** Values above branches indicate bootstrap values. The scale bar denotes the estimated number of nucleotide substitution per site. Bars on the right represent the A+T percentage and are proportional to those in Figure 1.
(TIF)

Supporting Information S4 **Phylogeny based on 24 mitochondrial/bacterial proteins inferred by Maximum Likelihood using standard amino acid coding and the WAG+F+Γ4 model.** Values above branches indicate bootstrap values. The scale bar denotes the estimated number of amino acid substitution per site.
(TIF)

Supporting Information S5 **Phylogeny based on 24 mitochondrial/bacterial proteins inferred by Maximum Likelihood using the Dayhoff coding of amino acids in six categories and the GTR+F+Γ4 model.** Values above branches indicate bootstrap values. The scale bar denotes the estimated number of amino acid substitution per site.
(TIF)

Supporting Information S6 **Phylogeny based on 25 mitochondrial/bacterial proteins inferred by Bayesian Inference using the WAG+F+Γ4 model with only HIMB59 to represent the SAR11 group.** Values above branches indicate posterior probabilities values. The scale bar denotes the estimated number of amino acid substitution per site.
(TIF)

Supporting Information S7 **Phylogeny based on 24 mitochondrial/bacterial proteins inferred by Bayesian Inference using the CAT mixture model with only HIMB59 to represent the SAR11 group.** Values above branches indicate posterior probabilities. The scale bar denotes the estimated number of amino acid substitution per site.
(TIF)

Supporting Information S8 **Phylogeny based on 24 mitochondrial/bacterial proteins inferred by Bayesian Inference using the Dayhoff recoded dataset and WAG+F+Γ4 model with only HIMB59 to represent the SAR11 group.** Values above branches indicate posterior probabilities values. The scale bar denotes the estimated number of amino acid substitution per site.
(TIF)

Supporting Information S9 **Phylogeny based on 24 mitochondrial/bacterial proteins inferred by Bayesian Inference using the Dayhoff recoded dataset and the CAT mixture model with only HIMB59 to represent the SAR11 group.** Values above branches indicate posterior probabilities. The scale bar denotes the estimated number of amino acid substitution per site.
(TIF)

Supporting Information S10 **Phylogeny based on 24 mitochondrial/bacterial proteins inferred by Maximum Likelihood using standard amino acid coding and the WAG+F+Γ4 model when a low %AT (left) or high %AT (right) outgroup is used.** Values above branches indicate bootstrap values. The scale bar denotes the estimated number of amino acid substitution per site.
(TIF)

Supporting Information S11 **Phylogeny based on 24 mitochondrial/bacterial proteins inferred by Maximum Likelihood using standard amino acid coding and the GTR+F+Γ4 model applied on the Dayhoff recoded datastet when a low %AT (left) or high %AT (right) outgroup is used.** Values above branches indicate bootstrap values. The scale bar denotes the estimated number of amino acid substitution per site.
(TIF)

Supporting Information S12 **Complete names and distribution of missing data among the species used.**
(DOC)

Supporting Information S13 **Protein coding genes used.**
(DOC)

Supporting Information S14 **Details of the Phylobayes analyses.** For each model and dataset, 2 independent chains were run and compared to assess convergence by computing the largest discrepancy across bipartitions (maxdiff). A maxdiff <0.3 is indicator of a good run.
(DOC)

Supporting Information S15 **Cross validation tests performed as implemented in PhyloBayes 3.3.** Mean score differences and standard deviation to the best fitting model are shown. Cross validation tests indicate that for both, the non-recoded and recoded datasets the CAT model is the one that has the best fit to the data.
(DOC)

Supporting Information S16 **Posterior predictive tests for saturation as implemented in Phylobayes.** The observed and predicted distributions of the saturation index summarised by their means and variances are indicated for each combination of dataset and model.
(DOC)

Supporting Information S17 **Posterior predictive tests for compositional homogeneity as implemented in Phylobayes.** The maximum deviation across taxa was calculated for the original non recoded and recoded datasets. For each dataset and model, replicates were simulated using the parameters of 1/100 of the sample points and the mean of the maximum deviation over the taxa was calculated for all replicates.
(DOC)
