# Supplementary figures and images for: The SAR11 Group of Alpha-Proteobacteria Is Not Related to the Origin of Mitochondria
Source: PLoS One. 2012 Jan 23;7(1):e30520. doi: 10.1371/journal.pone.0030520 (PMC3264578; doi:10.1371/journal.pone.0030520)

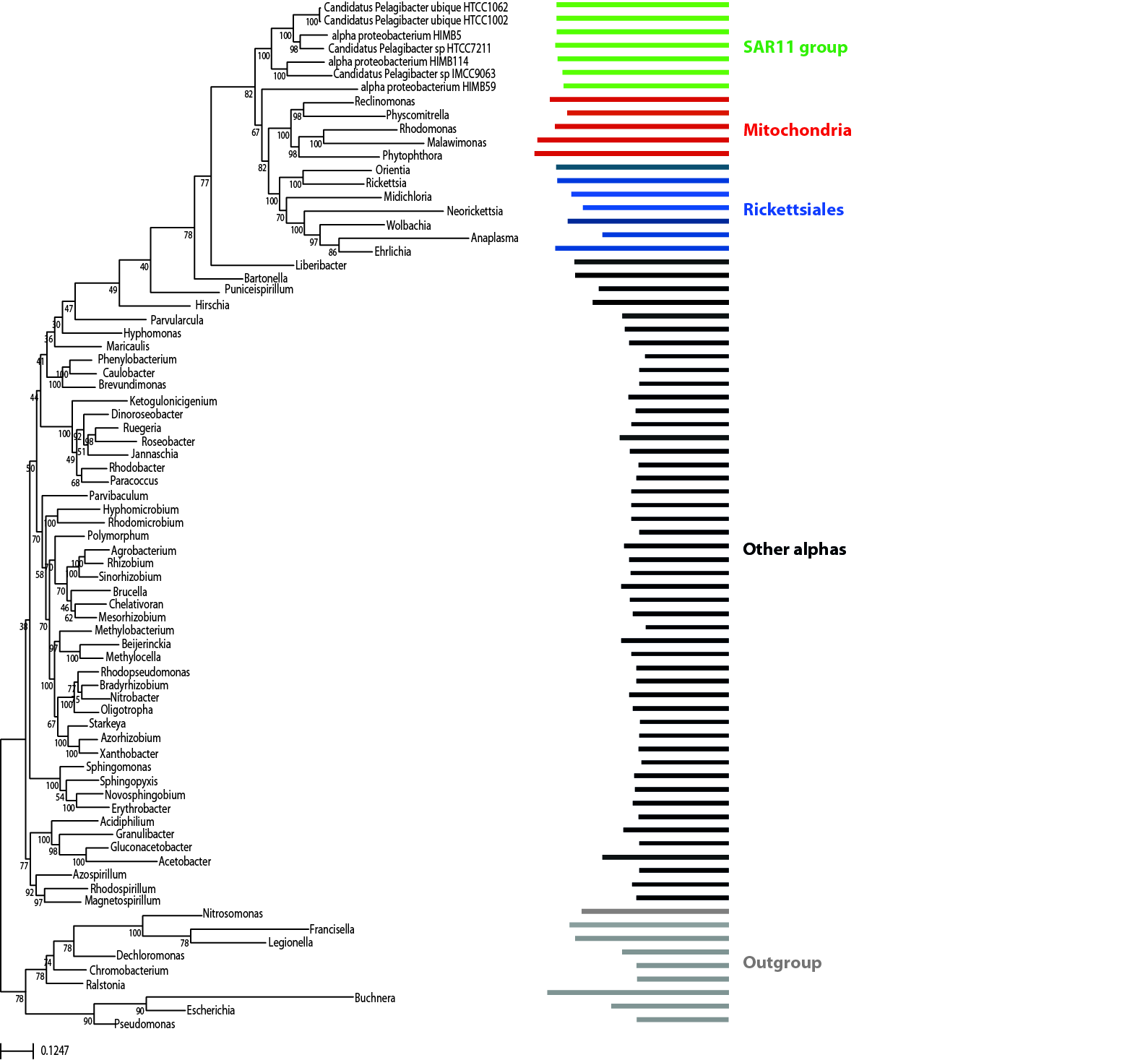

Supplement: Supporting Information S1 — Phylogeny based on 24 mitochondrial/bacterial protein coding genes inferred by Maximum Likelihood using GTR+F+Γ4 model. Values above branches indicate bootstrap values. The scale bar denotes the estimated number of nucleotide substitution per site. Bars on the right represent the A+T percentage and are proportional to those in Figure 1. (TIF) [file pone.0030520.s001.tif]

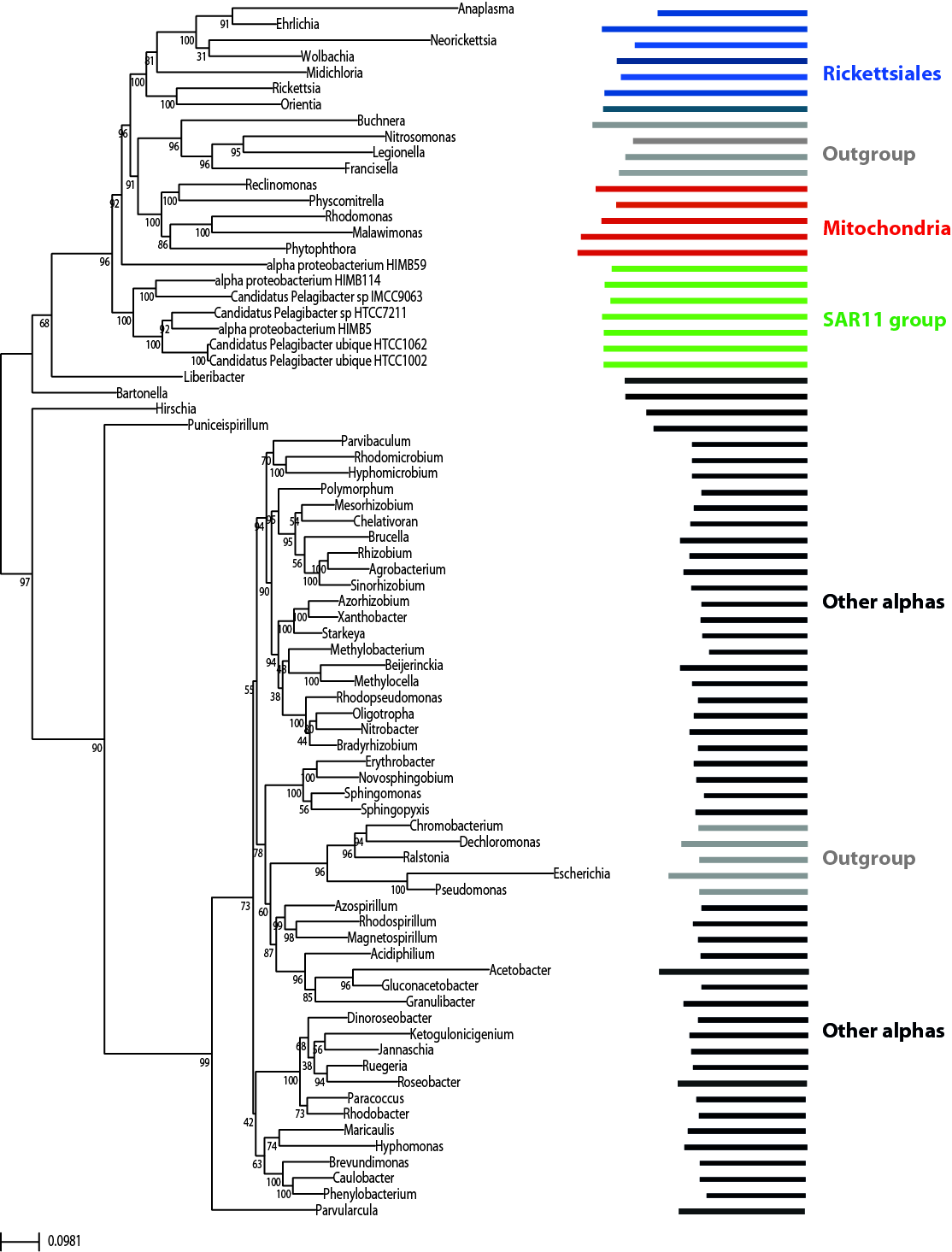

Supplement: Supporting Information S2 — Phylogeny based on 24 mitochondrial/bacterial protein coding genes excluding the third codon position inferred by Maximum Likelihood using GTR+F+Γ4 model. Values above branches indicate bootstrap values. The scale bar denotes the estimated number of nucleotide substitution per site. Bars on the right represent the A+T percentage and are proportional to those in Figure 1. (TIF) [file pone.0030520.s002.tif]

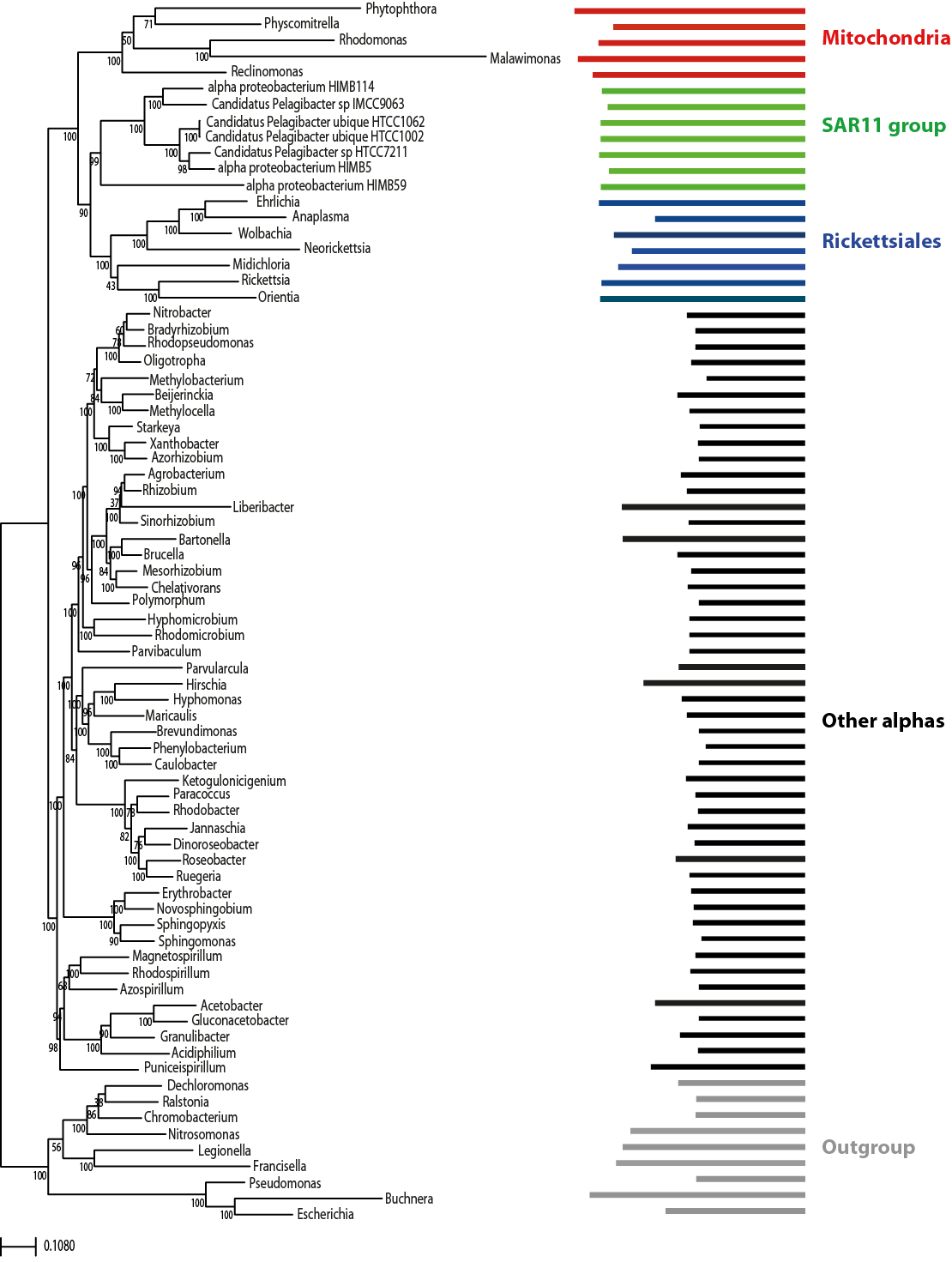

Supplement: Supporting Information S3 — Phylogeny based on 24 mitochondrial/bacterial protein coding genes inferred by Maximum Likelihood using the RY coding and the GTR+F+Γ4 model. Values above branches indicate bootstrap values. The scale bar denotes the estimated number of nucleotide substitution per site. Bars on the right represent the A+T percentage and are proportional to those in Figure 1. (TIF) [file pone.0030520.s003.tif]

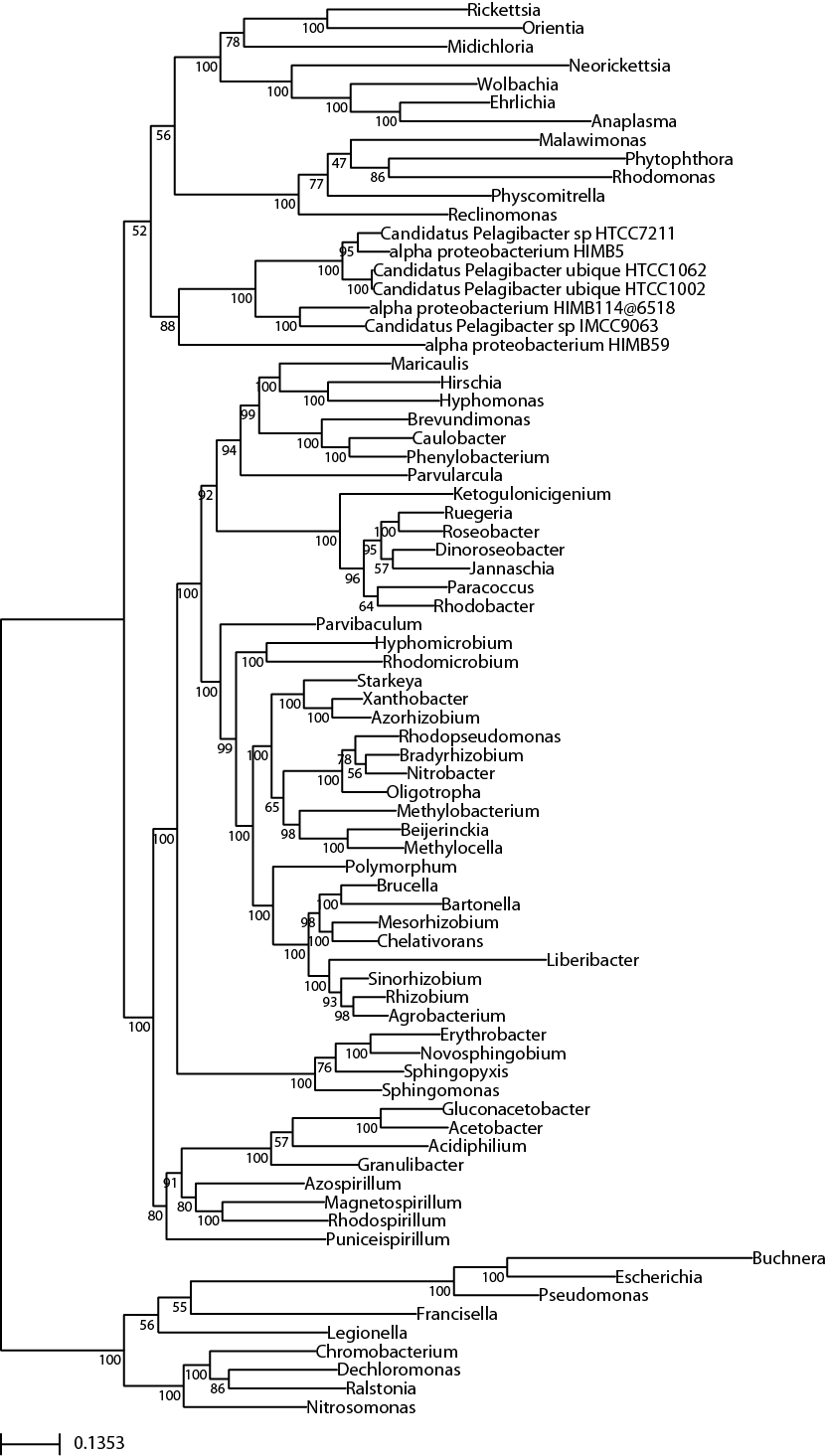

Supplement: Supporting Information S4 — Phylogeny based on 24 mitochondrial/bacterial proteins inferred by Maximum Likelihood using standard amino acid coding and the WAG+F+Γ4 model. Values above branches indicate bootstrap values. The scale bar denotes the estimated number of amino acid substitution per site. (TIF) [file pone.0030520.s004.tif]

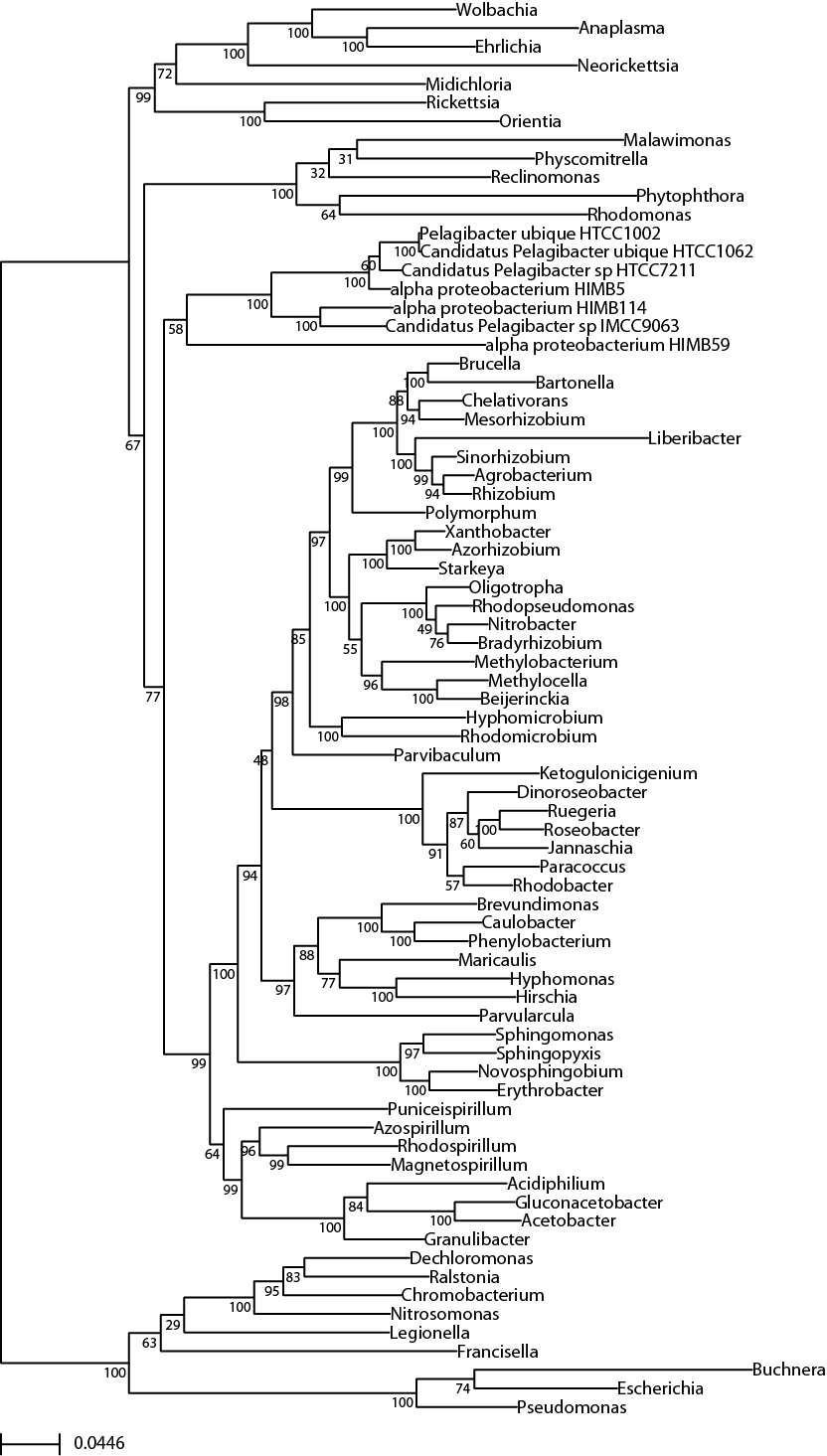

Supplement: Supporting Information S5 — Phylogeny based on 24 mitochondrial/bacterial proteins inferred by Maximum Likelihood using the Dayhoff coding of amino acids in six categories and the GTR+F+Γ4 model. Values above branches indicate bootstrap values. The scale bar denotes the estimated number of amino acid substitution per site. (TIF) [file pone.0030520.s005.tif]

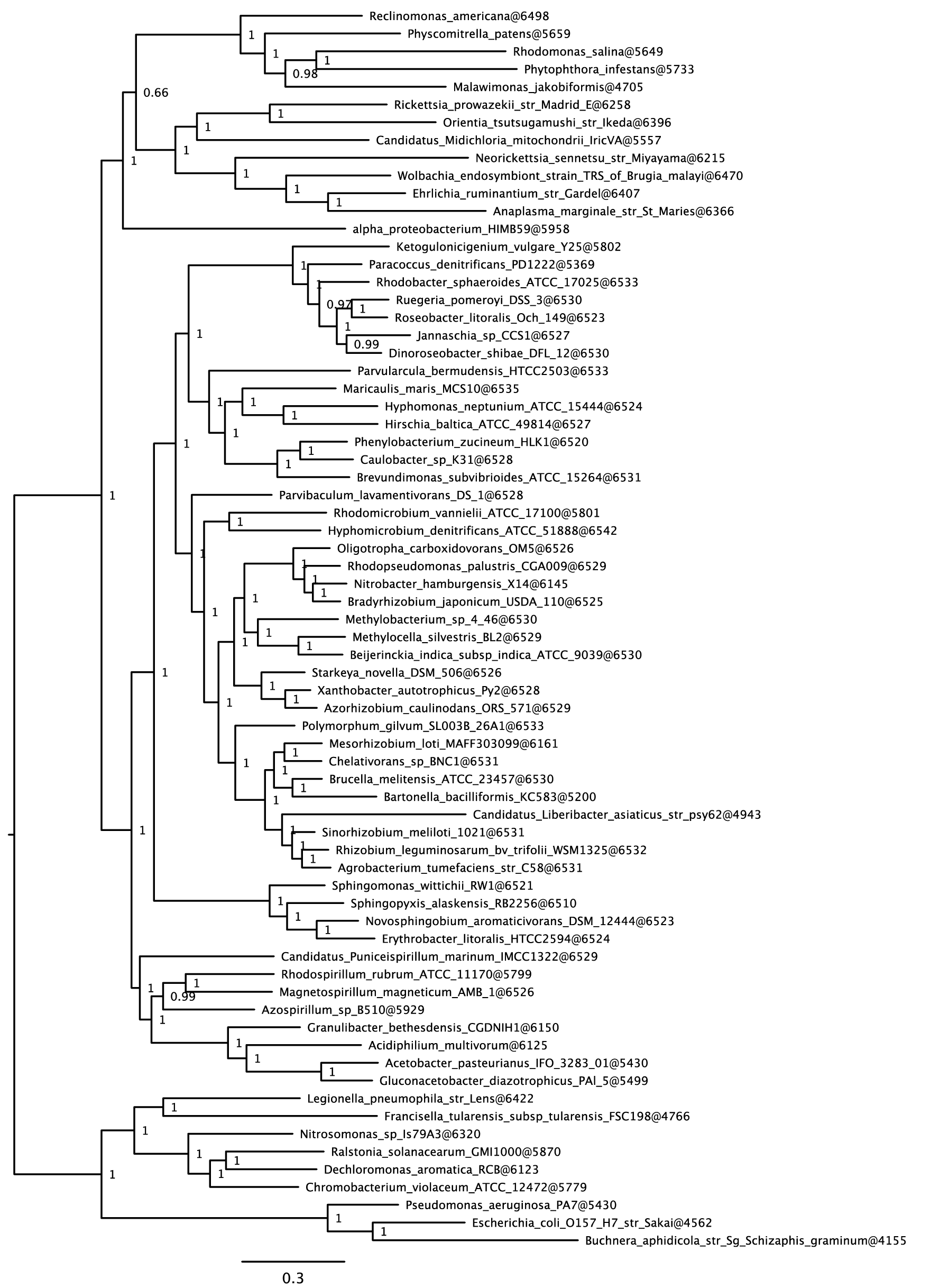

Supplement: Supporting Information S6 — Phylogeny based on 25 mitochondrial/bacterial proteins inferred by Bayesian Inference using the WAG+F+Γ4 model with only HIMB59 to represent the SAR11 group. Values above branches indicate posterior probabilities values. The scale bar denotes the estimated number of amino acid substitution per site. (TIF) [file pone.0030520.s006.tif]

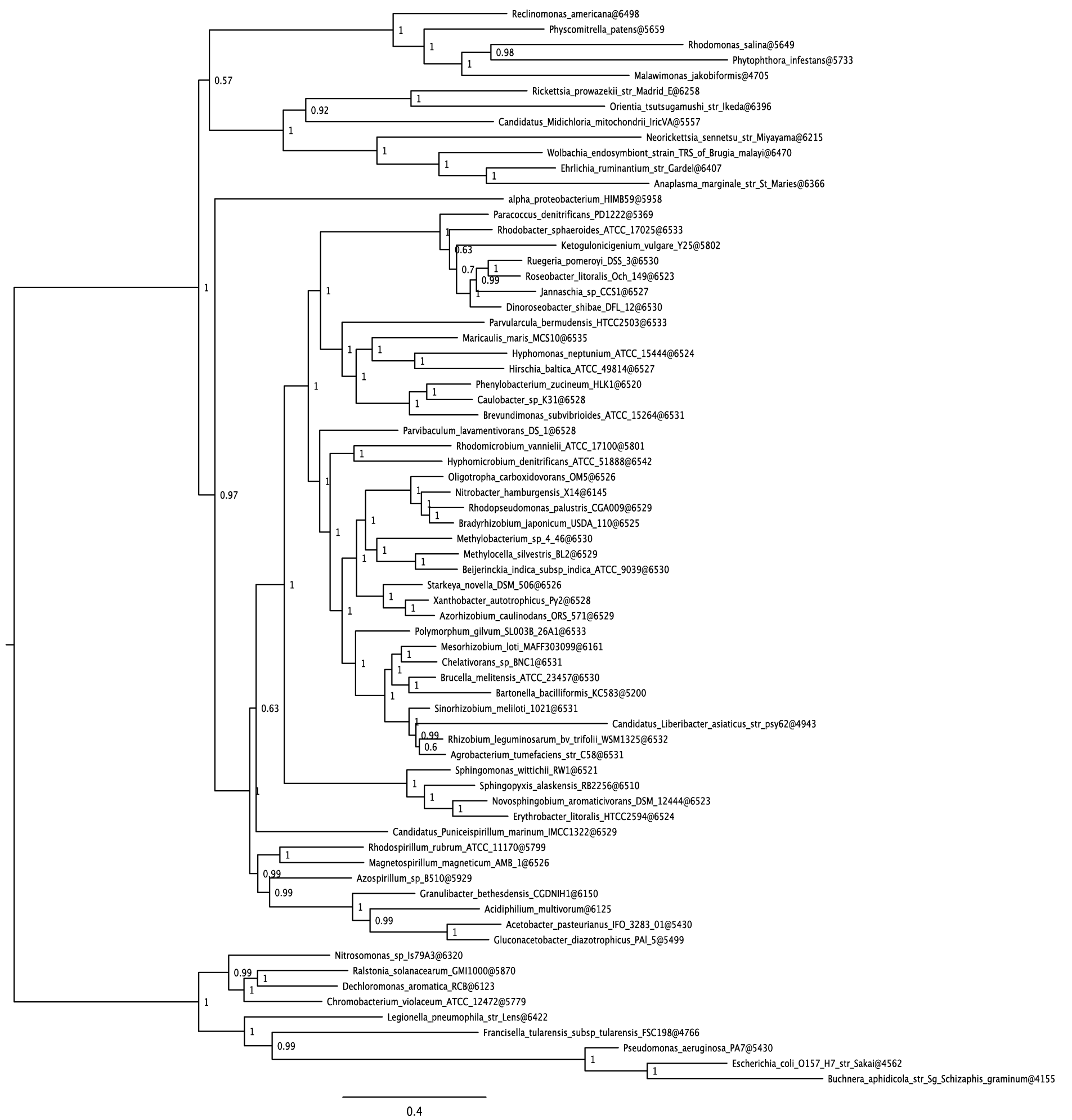

Supplement: Supporting Information S7 — Phylogeny based on 24 mitochondrial/bacterial proteins inferred by Bayesian Inference using the CAT mixture model with only HIMB59 to represent the SAR11 group. Values above branches indicate posterior probabilities. The scale bar denotes the estimated number of amino acid substitution per site. (TIF) [file pone.0030520.s007.tif]

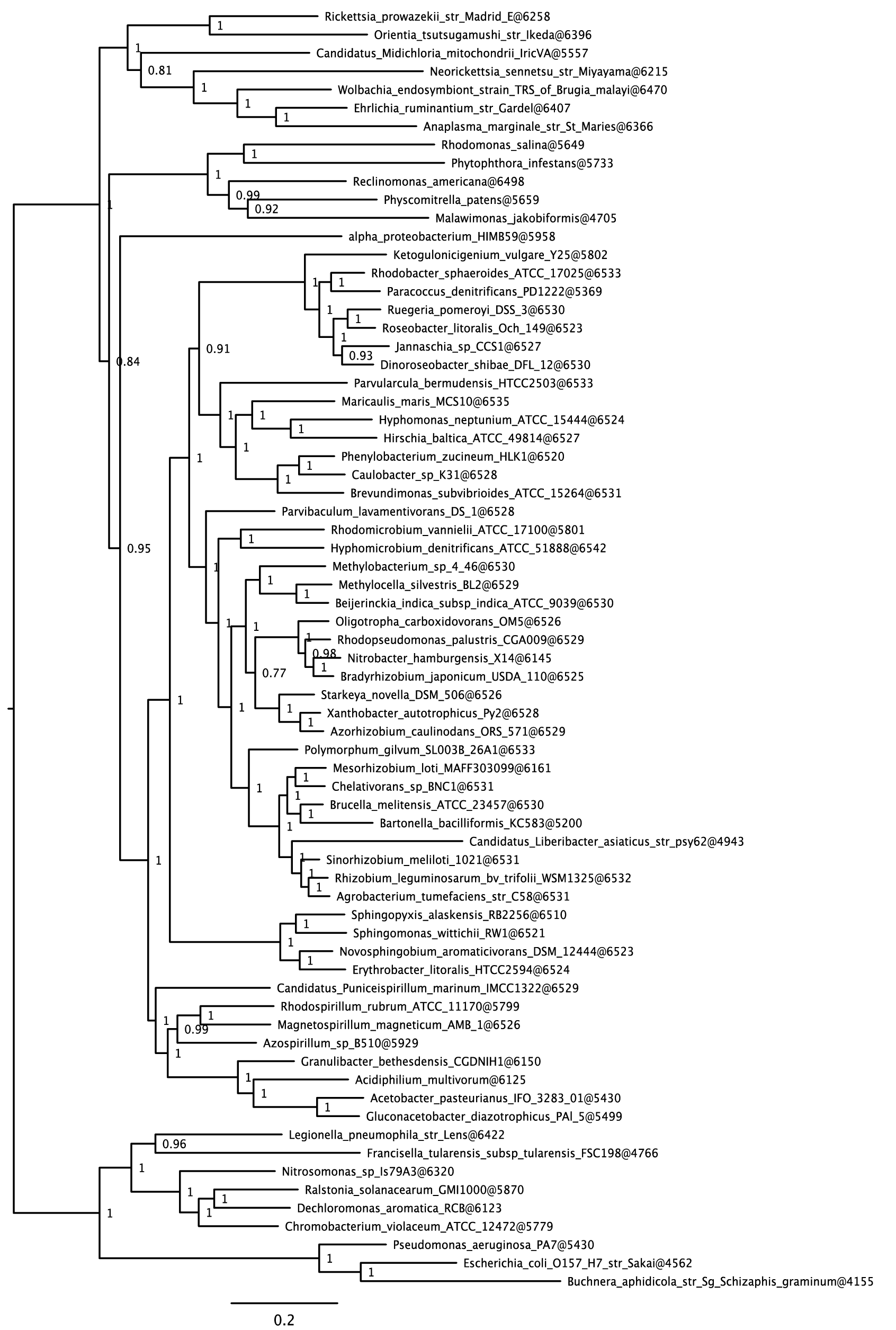

Supplement: Supporting Information S8 — Phylogeny based on 24 mitochondrial/bacterial proteins inferred by Bayesian Inference using the Dayhoff recoded dataset and WAG+F+Γ4 model with only HIMB59 to represent the SAR11 group. Values above branches indicate posterior probabilities values. The scale bar denotes the estimated number of amino acid substitution per site. (TIF) [file pone.0030520.s008.tif]

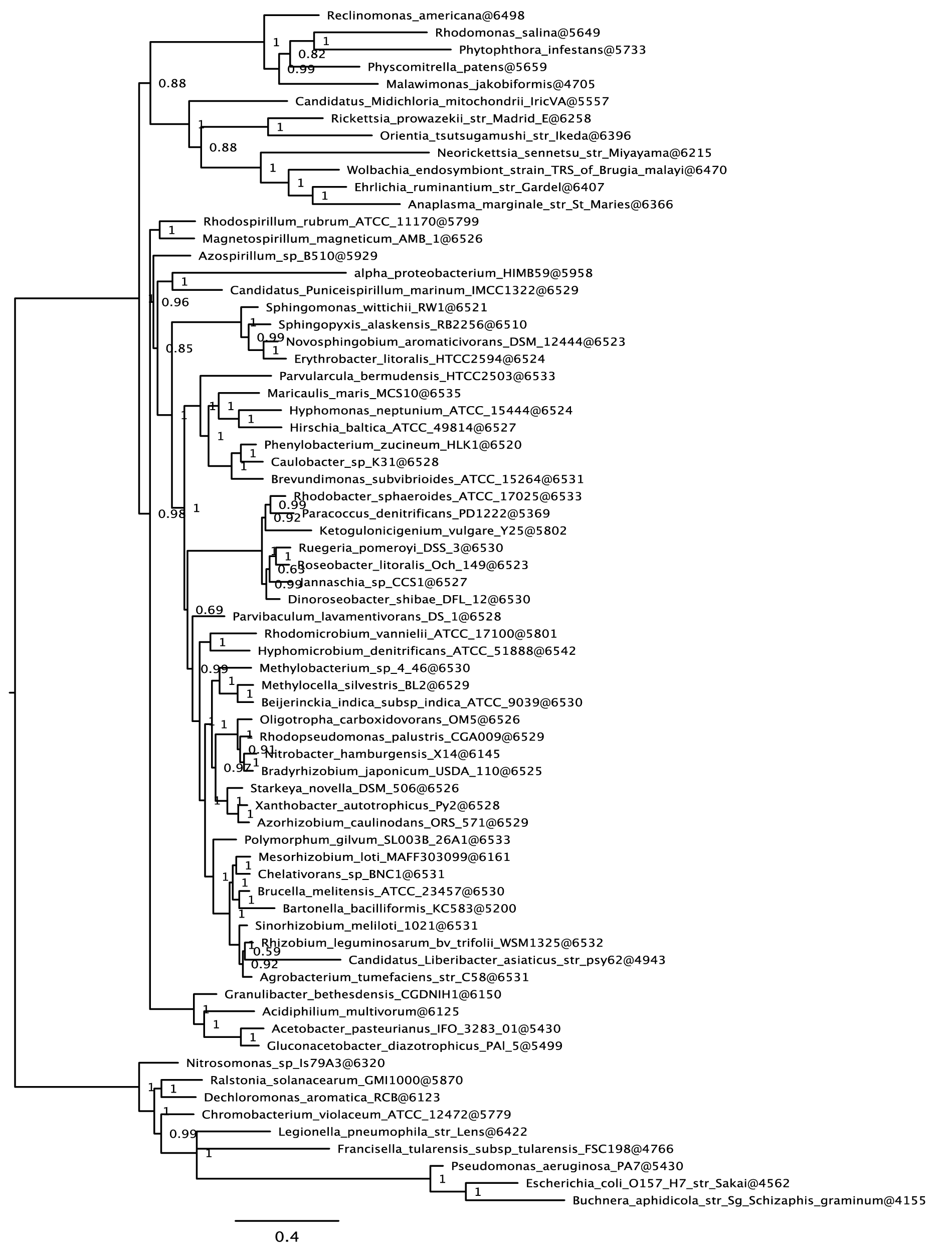

Supplement: Supporting Information S9 — Phylogeny based on 24 mitochondrial/bacterial proteins inferred by Bayesian Inference using the Dayhoff recoded dataset and the CAT mixture model with only HIMB59 to represent the SAR11 group. Values above branches indicate posterior probabilities. The scale bar denotes the estimated number of amino acid substitution per site. (TIF) [file pone.0030520.s009.tif]

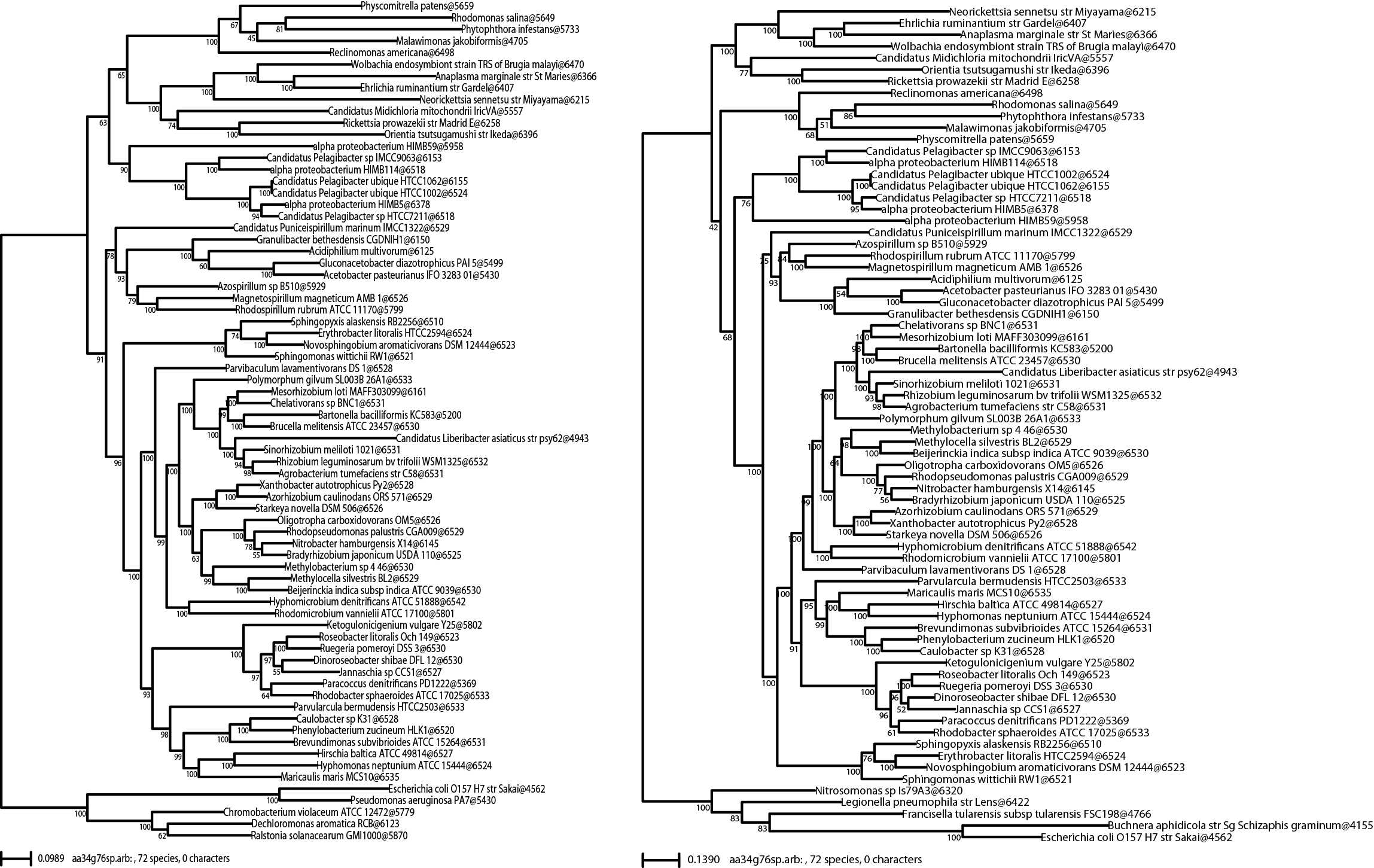

Supplement: Supporting Information S10 — Phylogeny based on 24 mitochondrial/bacterial proteins inferred by Maximum Likelihood using standard amino acid coding and the WAG+F+Γ4 model when a low %AT (left) or high %AT (right) outgroup is used. Values above branches indicate bootstrap values. The scale bar denotes the estimated number of amino acid substitution per site. (TIF) [file pone.0030520.s010.tif]

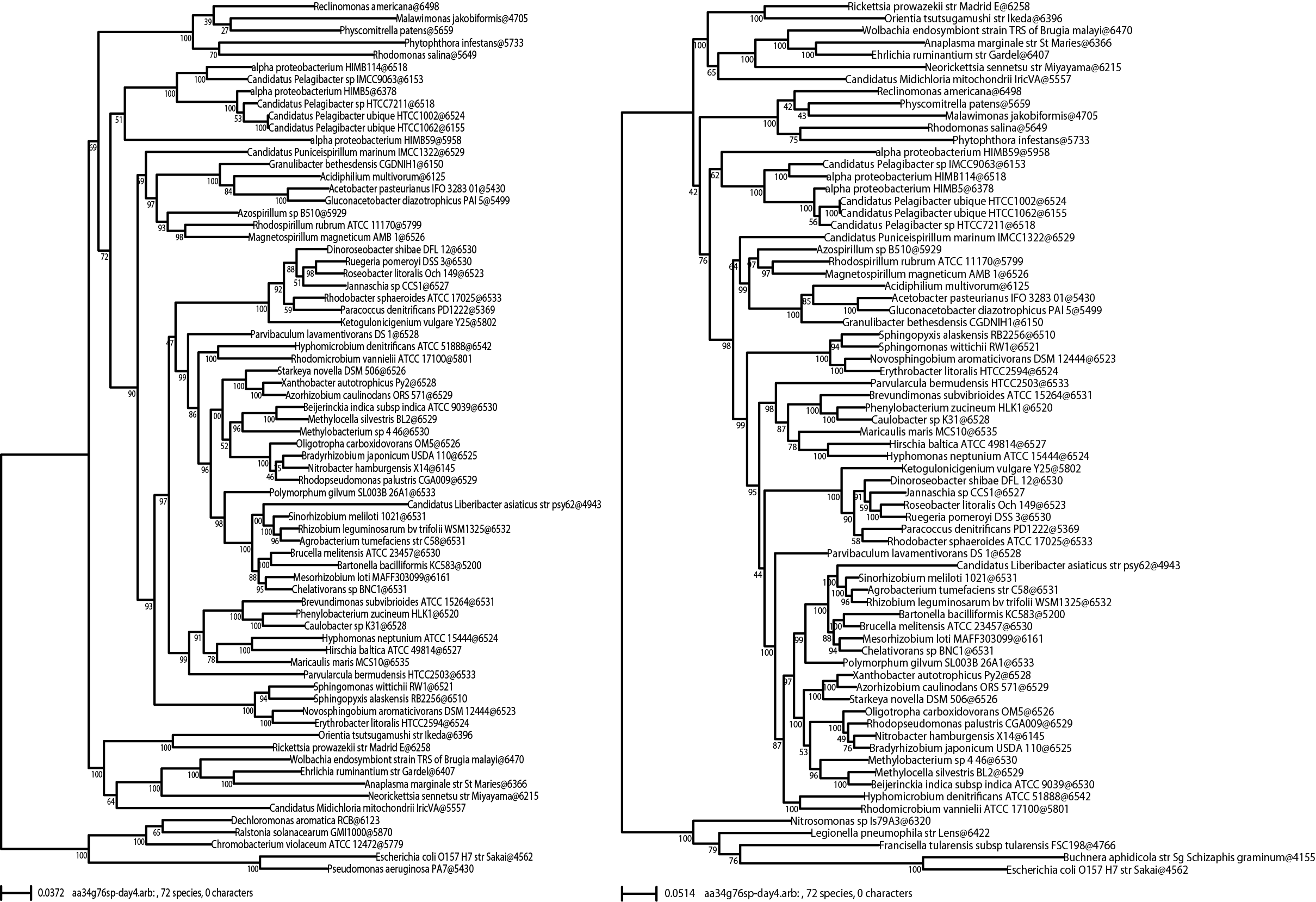

Supplement: Supporting Information S11 — Phylogeny based on 24 mitochondrial/bacterial proteins inferred by Maximum Likelihood using standard amino acid coding and the GTR+F+Γ4 model applied on the Dayhoff recoded datastet when a low %AT (left) or high %AT (right) outgroup is used. Values above branches indicate bootstrap values. The scale bar denotes the estimated number of amino acid substitution per site. (TIF) [file pone.0030520.s011.tif]
