# Supplementary material for: The SAR11 Group of Alpha-Proteobacteria Is Not Related to the Origin of Mitochondria
Source: PLoS One. 2012 Jan 23;7(1):e30520. doi: 10.1371/journal.pone.0030520 (PMC3264578; doi:10.1371/journal.pone.0030520)
Supplement: Supporting Information S12 — Complete names and distribution of missing data among the species used. (DOC) [file pone.0030520.s012.doc]

Table S1: Complete names and distribution of missing data among the species used.

| **Species Name** | **%missing** |
| --- | --- |
| *Acetobacter pasteurianus* IFO 3283 01 | 17 |
| *Acidiphilium multivorum* | 6 |
| *Agrobacterium tumefaciens str* C58 | 0 |
| *Anaplasma marginale str St Maries* | 2 |
| *Azorhizobium caulinodans* ORS 571 | 0 |
| *Azospirillum* sp B510 | 9 |
| *Bartonella bacilliformis* KC583 | 20 |
| *Beijerinckia indica* subsp indica ATCC 9039 | 0 |
| *Bradyrhizobium japonicum* USDA 110 | 0 |
| *Brevundimonas subvibrioides* ATCC 15264 | 0 |
| *Brucella melitensis* ATCC 23457 | 0 |
| *Buchnera aphidicola* str Sg Schizaphis graminum | 36 |
| *Candidatus* Liberibacter asiaticus str psy62 | 24 |
| *Candidatus* Midichloria mitochondrii IricVA | 15 |
| *Candidatus* Pelagibacter sp HTCC7211 | 0 |
| *Candidatus* Pelagibacter sp IMCC9063 | 6 |
| *Candidatus* Pelagibacter ubique HTCC1002 | 0 |
| *Candidatus* Pelagibacter ubique HTCC1062 | 6 |
| *Candidatus* Puniceispirillum marinum IMCC1322 | 0 |
| *Caulobacter* sp K31 | 0 |
| *Chelativorans* sp BNC1 | 0 |
| *Chromobacterium violaceum* ATCC 12472 | 11 |
| *Dechloromonas aromatica* RCB | 6 |
| *Dinoroseobacter shibae* DFL 12 | 0 |
| *Ehrlichia ruminantium* str Gardel | 2 |
| *Erythrobacter litoralis* HTCC2594 | 0 |
| *Escherichia coli* O157 H7 str Sakai | 30 |
| *Francisella tularensis* subsp tularensis FSC198 | 27 |
| *Gluconacetobacter diazotrophicus* PAl 5 | 16 |
| *Granulibacter bethesdensis* CGDNIH1 | 6 |
| *Hirschia baltica* ATCC 49814 | 0 |
| *Hyphomicrobium denitrificans* ATCC 51888 | 0 |
| *Hyphomonas neptunium* ATCC 15444 | 0 |
| *Jannaschia* sp CCS1 | 0 |
| *Ketogulonicigenium vulgare* Y25 | 11 |
| *Legionella pneumophila* str Lens | 2 |
| *Magnetospirillum magneticum* AMB 1 | 0 |
| *Malawimonas jakobiformis* | 27 |
| *Maricaulis maris* MCS10 | 0 |
| *Mesorhizobium loti* MAFF303099 | 6 |
| *Methylobacterium* sp 4 46 | 0 |
| *Methylocella silvestris* BL2 | 0 |
| *Neorickettsia sennetsu* str Miyayama | 5 |
| *Nitrobacter hamburgensis* X14 | 6 |
| *Nitrosomonas* sp Is79A3 | 3 |
| *Novosphingobium aromaticivorans* DSM 12444 | 0 |
| *Oligotropha carboxidovorans* OM5 | 0 |
| *Orientia tsutsugamushi* str Ikeda | 2 |
| *Paracoccus denitrificans* PD1222 | 17 |
| *Parvibaculum lavamentivorans* DS 1 | 0 |
| *Parvularcula bermudensis* HTCC2503 | 0 |
| *Phenylobacterium zucineum* HLK1 | 0 |
| *Physcomitrella patens* | 13 |
| *Phytophthora infestans* | 12 |
| *Polymorphum gilvum* SL003B 26A1 | 0 |
| *Pseudomonas aeruginosa* PA7 | 17 |
| *Ralstonia solanacearum* GMI1000 | 10 |
| *Reclinomonas americana* | 0 |
| *Rhizobium leguminosarum* bv trifolii WSM1325 | 0 |
| *Rhodobacter sphaeroides* ATCC 17025 | 0 |
| *Rhodomicrobium vannielii* ATCC 17100 | 11 |
| *Rhodomonas salina* | 13 |
| *Rhodopseudomonas palustris* CGA009 | 0 |
| *Rhodospirillum rubrum* ATCC 11170 | 11 |
| *Rickettsia prowazekii* str Madrid E | 4 |
| *Roseobacter litoralis* Och 149 | 0 |
| *Ruegeria pomeroyi* DSS 3 | 0 |
| *Sinorhizobium meliloti* 1021 | 0 |
| *Sphingomonas wittichii* RW1 | 0 |
| *Sphingopyxis alaskensis* RB2256 | 0 |
| *Starkeya novella* DSM 506 | 0 |
| *Wolbachia endosymbiont* strain TRS of Brugia malayi | 1 |
| *Xanthobacter autotrophicus* Py2 | 0 |
| alpha proteobacterium HIMB114 | 0 |
| alpha proteobacterium HIMB5 | 2 |
| alpha proteobacterium HIMB59 | 9 |
