# Supplementary material for: The SAR11 Group of Alpha-Proteobacteria Is Not Related to the Origin of Mitochondria
Source: PLoS One. 2012 Jan 23;7(1):e30520. doi: 10.1371/journal.pone.0030520 (PMC3264578; doi:10.1371/journal.pone.0030520)
Supplement: Supporting Information S13 — Protein coding genes used. (DOC) [file pone.0030520.s013.doc]

Table S2: Protein coding genes used.

| **Name** | **Length(aa)** |
| --- | --- |
| ribosomal protein S12 | 131 |
| NADH dehydrogenase subunit 11 | 691 |
| NADH dehydrogenase subunit 1 | 333 |
| cytochrome c oxidase subunit 3 | 267 |
| ribosomal protein S19 | 86 |
| ribosomal protein S3 | 287 |
| ribosomal protein L16 | 137 |
| ribosomal protein S14 | 100 |
| ribosomal protein S13 | 122 |
| ribosomal protein S4 | 249 |
| Sec-independent protein translocase component TatC | 260 |
| ATP synthase F1 subunit alpha | 512 |
| NADH dehydrogenase subunit 6 | 205 |
| NADH dehydrogenase subunit 7 | 396 |
| NADH dehydrogenase subunit 9 | 204 |
| NADH dehydrogenase subunit 3 | 122 |
| ABC transporter subunit C | 262 |
| NADH dehydrogenase subunit 4L | 102 |
| NADH dehydrogenase subunit 5 | 670 |
| NADH dehydrogenase subunit 4 | 493 |
| ribosomal protein S2 | 212 |
| ATP synthase F0 subunit 6 | 249 |
| Apocytochrome b | 390 |
| cytochrome c oxidase subunit 1 | 531 |
