# Supplementary material for: The SAR11 Group of Alpha-Proteobacteria Is Not Related to the Origin of Mitochondria
Source: PLoS One. 2012 Jan 23;7(1):e30520. doi: 10.1371/journal.pone.0030520 (PMC3264578; doi:10.1371/journal.pone.0030520)
Supplement: Supporting Information S14 — Details of the Phylobayes analyses. For each model and dataset, 2 independent chains were run and compared to assess convergence by computing the largest discrepancy across bipartitions (maxdiff). A maxdiff <0.3 is indicator of a good run. (DOC) [file pone.0030520.s014.doc]

Table S3: Details of the Phylobayes analyses. For each model and dataset, 2 independent chains were run and compared to assess convergence by computing the largest discrepancy across bipartitions (maxdiff). A maxdiff < 0.3 is indicator of a good run.

| Dataset | **Model** | **Chain length** | **Burn-in length** | **maxdiff** | **meandiff** |
| --- | --- | --- | --- | --- | --- |

**All species**

| Non recoded dataset | CAT | 3 10^6^ | 5000 | 0.04 | 0.0005 |
| --- | --- | --- | --- | --- | --- |
|  | WAG | 4.5 10^5^ | 50 | 0.08 | 0.0003 |
| Dayhoff6 recoded dataset | CAT | 1.8 10^6^ | 500 | 0.06 | 0.0016 |
|  | GTR | 1.1 10^6^ | 50 | 0.08 | 0.0004 |

**Without HIMB59**

| Non recoded dataset | CAT | 1.7 10^6^ | 3000 | 0.05 | 0.0006 |
| --- | --- | --- | --- | --- | --- |
|  | WAG | 4.5 10^5^ | 150 | 0.02 | 0.0001 |
| Dayhoff6 recoded dataset | CAT | 1.7 10^6^ | 500 | 0.08 | 0.0013 |
|  | GTR | 5 10^5^ | 100 | 0.02 | 0.0002 |

**Only HIMB59**

| Non recoded dataset | CAT | 1.5 10^6^ | 3000 | 0.20 | 0.0020 |
| --- | --- | --- | --- | --- | --- |
|  | WAG | 4 10^5^ | 150 | 0.04 | 0.0003 |
| Dayhoff6 recoded dataset | CAT | 1 10^6^ | 500 | 0.07 | 0.0011 |
|  | GTR | 4 10^5^ | 50 | 0.03 | 0.0005 |
