# Supplementary material for: The SAR11 Group of Alpha-Proteobacteria Is Not Related to the Origin of Mitochondria
Source: PLoS One. 2012 Jan 23;7(1):e30520. doi: 10.1371/journal.pone.0030520 (PMC3264578; doi:10.1371/journal.pone.0030520)
Supplement: Supporting Information S15 — Cross validation tests performed as implemented in PhyloBayes 3.3. Mean score differences and standard deviation to the best fitting model are shown. Cross validation tests indicate that for both, the non-recoded and recoded datasets the CAT model is the one that has the best fit to the data. (DOC) [file pone.0030520.s015.doc]

Table S4: Cross validation tests performed as implemented in PhyloBayes 3.3. Mean score differences and standard deviation to the best fitting model are shown. Cross validation tests indicate that for both, the non-recoded and recoded datasets the CAT model is the one that has the best fit to the data.

| Dataset | **Model** | **Mean score difference** | **Standard Deviation** |
| --- | --- | --- | --- |
| Non recoded amino acid dataset | CAT | 0 | +/- 0 |
|  | WAG | -1318. 6 | +/- 117.46 |
| Dayhoff6 recoded amino acid dataset | CAT | 0 | +/- 0 |
|  | GTR | -1388.47 | +/- 84.471 |
