# Supplementary material for: The SAR11 Group of Alpha-Proteobacteria Is Not Related to the Origin of Mitochondria
Source: PLoS One. 2012 Jan 23;7(1):e30520. doi: 10.1371/journal.pone.0030520 (PMC3264578; doi:10.1371/journal.pone.0030520)
Supplement: Supporting Information S16 — Posterior predictive tests for saturation as implemented in Phylobayes. The observed and predicted distributions of the saturation index summarised by their means and variances are indicated for each combination of dataset and model. (DOC) [file pone.0030520.s016.doc]

Table S5: Posterior predictive tests for saturation as implemented in Phylobayes. The observed and predicted distributions of the saturation index summarised by their means and variances are indicated for each combination of dataset and model.

| Dataset | **Model** | **observed homoplasy** | **predicted homoplasy** |
| --- | --- | --- | --- |
| Non recoded dataset | CAT | 20.4849  (+/- 0.787622) | 20.3547  (+/- 0.806035) |
|  | WAG | 16.2693  (+/- 0.0736124) | 14.7644  (+/- 0.159854) |
| Dayhoff6 recoded dataset | CAT | 15.2207  (+/- 2.4408) | 15.2755  (+/- 2.45137) |
|  | GTR | 10.3247  (+/- 0.060392) | 9.8637  (+/- 0.097146) |
