# Supplementary material for: The SAR11 Group of Alpha-Proteobacteria Is Not Related to the Origin of Mitochondria
Source: PLoS One. 2012 Jan 23;7(1):e30520. doi: 10.1371/journal.pone.0030520 (PMC3264578; doi:10.1371/journal.pone.0030520)
Supplement: Supporting Information S17 — Posterior predictive tests for compositional homogeneity as implemented in Phylobayes. The maximum deviation across taxa was calculated for the original non recoded and recoded datasets. For each dataset and model, replicates were simulated using the parameters of 1/100 of the sample points and the mean of the maximum deviation over the taxa was calculated for all replicates. (DOC) [file pone.0030520.s017.doc]

Table S6: Posterior predictive tests for compositional homogeneity as implemented in Phylobayes. The maximum deviation across taxa was calculated for the original non recoded and recoded datasets. For each dataset and model, replicates were simulated using the parameters of 1/100 of the sample points and the mean of the maximum deviation over the taxa was calculated for all replicates.

| Dataset | **Model** | **Max observed deviation** | **Mean Max predicted deviation** | **Z score** | **p value** |
| --- | --- | --- | --- | --- | --- |
| Non recoded dataset | CAT | 0.0107831 | 0.00093 | 13.55 | 0 |
|  | WAG | 0.0107831 | 0.00067 | 16.24 | 0 |
| Dayhoff6 recoded dataset | CAT | 0.0102297 | 0.00145 | 11.35 | 0 |
|  | GTR | 0.0102297 | 0.00102 | 8.903 | 0 |
